# Experiences of patients and public partners in codesign of Lynch Choices™: an evaluation study using the Patient Engagement In Research Scale (PEIRS-22)

**DOI:** 10.1186/s40900-026-00854-z

**Published:** 2026-03-09

**Authors:** Kelly Kohut, Lesley Turner, Caroline Dale, Sue Duncombe, Rochelle Gold, Sonia Patton, Richard Stephens, Frankie Vale, Helen White, Steve Worrall, Julie Young, Tracy Smith, Roberta Horgan, Kate Morton, Becky Foster, the International Lynch Decision Aid Expert Partner Panel, Munaza Ahmed, Lyndsy Ambler, Antonis Antoniou, Stephanie Archer, Ruth Armstrong, Elizabeth Bancroft, Kristine Barlow-Stewart, Lily Barnett, Marion Bartlett, Julian Barwell, Dany Bell, Cheryl Berlin, Felicity Blair, Matilda Bradford, John Burn, Sarah Cable, Melissa Cambell-Kelly, Dharmisha Chauhan, Ruth Cleaver, Beth Coad, Gaya Connolly, Gillian Crawford, Emma Crosbie, Victoria Cuthill, Tabib Dabir, Mev Dominguez-Valentin, Eleanor Davies, Glyn Elwyn, Mary Jane Esplen, D Gareth Evans, Pia Fabricius, Andrea Forman, Kaisa Fritzell, Claire Giffney, Joana Gomes, Rebecca Hall, Helen Hanson, Menna Hawkins, Deborah Holliday, Roberta Horgan, Karen Hurley, Margaret James, Ros Jewell, Siobhan John, Victoria Kiesel, Anna Koziel, Anjana Kulkarni, Fiona Lalloo, Helen Liggett, Aela Limbu, Kate Lippiett, Anne Lowry, Manami Matsukawa, Ranjit Manchanda, Tracie Miles, Shakira Milton, Pål Møller, Kevin Monahan, Laura Monje-Garcia, Gabriela Moslein, Alex Murray, Jennie Murray, Kai-Ren Ong, Anbu Paramasivam, Alison Pope, Sarah Pugh, Imran Rafi, Gabriel Recchia, Nicola Reents, Neil Ryan, Sibel Saya, Raza Sayyed, Salma Shickh, Toni Seppala, Lucy Side, Katie Snape, Sian Smith, Tracy Smith, Dawn Stacey, Barbara Stayner, Eriko Takamine, Katrina Tatton-Brown, Helle Vendel Petersen, Robert Volk, Karen Westaway, Nikki Warner, Jennifer Wiggins, Lisa Wilde, Jennet Williams, Catherine Willis, Elizabeth Winchester, Emma Woodward, Alice Youngs, Diana Eccles, Claire Foster

**Affiliations:** 1 Department of Clinical and Biomedical Sciences, University of Exeter Medical School, Exeter, UK; 2 Centre for Psychosocial Research in Cancer: CentRIC, School of Health Sciences, University of Southampton, Southampton, UK; 3 South West Thames Centre for Genomics, St. George’s University Hospitals NHS Foundation Trust, London, UK; 4 CanGene-CanVar Patient Reference Panel, Southampton, UK; 5 Lynch Syndrome UK, Cardiff, UK; 6 Lynch Syndrome Ireland, Dublin, Ireland; 7 Faculty of Medicine, University of Southampton, Southampton, UK

**Keywords:** Patient and public involvement and engagement, Codesign, Coproduction, Codevelopment, Research partnerships, Patient engagement in research scale

## Abstract

**Background:**

Patient and public involvement and engagement (PPIE) in health research aims to make research more relevant, clear and useful to deliver results that matter most to people. PPIE can be valuable but it is not always easy. It is not yet part of every project. This paper shares a case study and review of PPIE in the Lynch Choices™ (https://canchoose.org.uk) co-design project. It adds to the evidence on good practice for PPIE in research.

**Methods:**

Patient Panel members and community partners completed the Patient Engagement in Research Scale (PEIRS-22). This short survey asked about practical arrangements, ease of taking part, contributions, teamwork, support, feeling valued and expected benefits. Each question was scored on a 5-point scale. We calculated scores for each person and the group. People also wrote personal stories about their experience. A researcher did the same. These stories gave deeper insight into feelings, ideas and experiences the survey might miss. They explained why people felt as they did and highlighted areas for improvement. We then looked for key themes in the stories using deductive and some inductive coding.

**Results:**

Ten out of 12 Patient Panel members, one Trustee of Lynch Syndrome UK, and one Trustee of Lynch Syndrome Ireland took part (12/14=86% response rate). The average score was 89 out of 100. The middle score was 93 out of 100, above the level called “very meaningful engagement”. Most scores were high in all areas. Quotes from the stories were grouped into seven themes, based on the PEIRS framework.

**Conclusion:**

This case study shows how trust, open two-way communication and strong working relationships helped make the Lynch Choices™ project a success. Mostly positive feedback was linked to people feeling valued and part of the team. Building these relationships took time. Contributors said that being part of the project was rewarding, enjoyable and motivating. Most suggested the project outputs would help others in practical and meaningful ways. This study adds to the evidence on PPIE in research, with recommendations for how to improve in future. PPIE should be adequately funded and planned from the start of projects. Two-way learning helps to share benefits with the people the research aims to help.

**Supplementary Information:**

The online version contains supplementary material available at 10.1186/s40900-026-00854-z.

## Abstract (plain language summary)

This paper describes patient and public involvement and engagement (PPIE) in research. We partnered with patients, researchers and charities to codesign a decision aid about managing genetic cancer risks, called Lynch Choices. We asked partners to complete a short survey called the Patient Engagement in Research Scale (PEIRS-22), to understand how everyone felt about being involved. The survey measures things like how easy it was to take part and if people felt listened to and part of the team. We also invited people to share personal stories, so we could hear ideas and feelings that the survey might miss. We invited 12 patient panel members and two charity partners to complete the survey. Twelve of them did and shared their stories too. Most feedback was positive. The total PEIRS-22 score was high. People liked clear communication, feeling like part of a true team with researchers, and seeing how their ideas could help others. The personal stories gave extra detail about why being involved mattered and how it boosted confidence and sense of purpose. Some suggestions for improvement were faster payments, more regular updates and involving partners to write papers in their own words. Key lessons learnt from partners were that building trust takes time and keeping honest, two-way communication is essential. We recommend that future researchers include PPIE to really learn from people. They should plan PPIE from the start. They should set aside enough funding and clearly define roles. This will add value to their research and help to share the benefits with the people it aims to help.

**Supplementary Information:**

The online version contains supplementary material available at 10.1186/s40900-026-00854-z.

## Background

Patient and public involvement (PPI) is encouraged and increasingly required by research funders [1]. Patients are experts in their own lives, with decision-making shaped by their social interactions and healthcare experiences [2]. They and other public partners can contribute across all research phases, ideally from project conception [3]. They may help to shape questions, review study documents, generate points to consider in research design. Once the study is completed, they can assist with wider dissemination of findings and outputs through their networks [4, 5]. Roles may include co-lead, co-investigators or co-author, depending on engagement level.

PPIE aims include improving research quality, relevance, and transparency by aligning with patient priorities. Despite UK guidance and evidence of benefits [1, 6–8], challenges such as limited funding, time, and researcher skills persist. Barriers include institutional and individual factors [9], sometimes resulting in tokenistic involvement [10]. This can leave contributors feeling isolated or undervalued [3, 11]. Trust is critical for meaningful engagement [12] and systematic reviews confirm benefits but highlight variability, requiring tailored strategies from the outset [13]. Training and peer support could help to enhance confidence and inclusivity [14].

The Health Research Authority audited 10% of almost 4000 studies seeking ethical approval and reported that 74% (296/398) reported PPIE, including 88/165 (53%) commercial studies and 208/233 (89%) non-commercial studies [11]. This is encouraging, but continued efforts are needed. International guidance has improved reporting quality; a review of 65 frameworks found no single best fit, recommending codesigned approaches [15]. The GRIPP2 (Guidance for Reporting Involvement of Patients and the Public) checklist supports transparent reporting [16]. Six UK standards - inclusive opportunities, working together, support and learning, communications, impact and governance - provide benchmarks [17], though uptake remains inconsistent [18].

Codesign (or cocreation, coproduction) promotes power-sharing partnerships [19–23] and is useful for developing complex health interventions [24, 25]. A meta-analysis of 26 international studies supported its effectiveness; however, more research is needed on evaluation [26]. Cost and complexity remain concerns [27]. Barriers include poor communication, time constraints and preconceived assumptions [28], alongside ethical issues such as conflicts or confidentiality breaches, which codesigned frameworks can help address [29].

Measuring meaningful PPIE is essential for learning and improvement [16, 30]. To address the lack of quantitative tools, Hamilton et al. (2018, 2021) developed the Patient Engagement in Research Scale (PEIRS-22) [5, 31], which assesses engagement quality across seven domains: procedural requirements, convenience, contributions, team environment, support, feeling valued, and benefits. Prioritising patient perspectives was a strength of the PEIRS-22 codesign project, though limited demographic diversity and the inclusion of only participants with arthritis may have led to similar experiences [31]. PEIRS-22 evaluates engagement quality from patients’ and public partners’ perspectives using a five-point Likert scale across seven domains: procedural requirements, convenience, contributions, team environment, support, feeling valued, and benefits [5, 31].

### Partnership between research team and patient reference panel

The research team (KK, KM, RF, CF, DE) partnered with a Patient Reference Panel (Chaired by LT, Members CD, SD, RG, SP, RS, JY, HW, SW) to codesign a new patient information hub and decision aid about inherited cancer risk management. The establishment and coordination of PPIE in the project has been described in detail in other publications [32, 33]. PPIE was included from conception of the research idea through completion of the literature review, interview studies and dissemination of the outputs through publications, presentations and mixed media platforms (duration more than five years, 2019–2025) [34–36]. The Patient Reference Panel included up to 12 people with a mix of personal history of cancer, family history of cancer and diagnosis of an inherited cancer predisposition (Lynch syndrome *n* = 5, BRCA *n* = 3). Ground rules and terms of reference were agreed upon at the beginning, with members free to leave at any time. Some were members from the conception of the project, whereas others joined later but were welcomed as equal contributors. Some remained engaged throughout, whereas others became less involved over time. This may have been due to other competing priorities or changes in circumstances, but it is also possible that their contact details changed and that the research team was not notified. Notice was given in advance of any online or in-person meetings, including opportunities to give preferences regarding date, time and agenda items. Although none of the Patient Reference Panel meetings included attendance by all members (n-12), there was enough representation at each one (generally 4–8 people) to have productive discussions that included varied views, perspectives and opinions. Those who were unable to attend were offered the opportunity to provide feedback by email, which was also elicited from public partners and other experts throughout the project.

### Expansion of partnerships to form an expert partner panel

Key public partners included the charities Lynch Syndrome UK (https://www.lynch-syndrome-uk.org, Trustee: TS) and Lynch Syndrome Ireland (https://lynchsyndromeireland.com, Trustee: RH) due to their close affiliation with the Lynch syndrome communities and national recognition as trusted leaders advocating for their members. TS and RH were asked to join the Expert Partner Panel, along with more than 100 other clinical and academic experts worldwide (see Expert Partner Panel names in acknowledgements and related publications. Note: the previous name of ‘Stakeholder’ was replaced by ‘Partner’) [32]. Partners were included by personal invitation by the researchers, based on their personal contacts and knowledge of experts in the field. The Expert Partner Panel received regular updates and request for feedback during the research study. This was usually by email from KK, with a few large or small online meetings where needed to discuss specific topics. Overall, the Expert Partner Panel included the Patient Reference Panel, TS from Lynch Syndrome UK, RH from Lynch Syndrome Ireland along with representatives from other patient groups, charities, clinical or academic institutions. Members could suggest other experts to invite. The group expanded during the project.

### PPIE activities in our research project

PPIE activities included online Patient Reference Panel meetings (*n* = 8), in-person meetings for the overarching research programme contributors and steering group (*n* = 4, annual) and periodic email communication (averaging about one group email every 3 months, with a varied number of additional emails between Patient Reference Panel, public and other partners in the interim period). Regular contact with patient and public partners elicited contributions that drove the direction of research and codesign of the decision aid. Partners were invited to review data analysis and reporting of the research studies and approved final versions of manuscripts before submission for publication. They also helped with the dissemination of outputs within their networks and on social media. Although most PPIE activities were virtual, partly due to the timing of the COVID-19 pandemic, some impactful in-person meetings were also included [37]. Personal communication suggested that these interactions helped build strong relationships and trust between partners and researchers.

### Timing and level of PPIE

The **ACTIVE framework** [38] was consulted to aid transparent reporting of the following constructs:

**Who was involved?** Patients and stakeholders.

**How were stakeholders recruited?** Open, flexible, by invitation.

**What was the mode of involvement?** Continuous approach, direct interaction methods.

**At what stage (and level) did the involvement occur?** Developing the research question (influencing), plan methods (co-leading, controlling), write protocol (contributing), develop research (contributing), run research (receiving), select studies (receiving), collect data (contributing), analyse data (contributing), interpret findings (influencing), write and publish (influencing), knowledge translation and impact (co-leading).

### Lynch choices™: a patient information hub and decision aid

The patient resource that was codesigned in this project is called Lynch Choices™: https://canchoose.org.uk [33]. This was iteratively codeveloped and refined via the person-based approach [39] including a systematic literature review [36], extensive PPIE and in-depth qualitative and quantitative research. Lynch Choices™ provides a trusted, central source of information with embedded evidence- and theory-based decision aids for Lynch syndrome carriers. Lynch syndrome is caused by a pathogenic variant (mutation) in one of four mismatch repair genes conferring gene-specific increased lifetime risks for certain cancers [40], mainly colorectal, endometrial and ovarian [41, 42]. UK management guidelines include recommendations for surveillance with two-yearly colonoscopy beginning at the age of 25–35 years, risk-reducing interventions and lifestyle modifications for primary prevention. Management guidelines are gene-, age- and sex-specific (https://www.ukcgg.org). A live link is included between Lynch Choices™ and the Prospective Lynch Syndromes Database (PLSD, https://plsd.eu) which displays personalised cancer risk penetrance estimates for Lynch syndrome carriers undergoing colonoscopy surveillance. Two primary cancer prevention decision aids are included, regarding the choices to take daily aspirin for at least two years to lower the risk of developing cancer [43, 44] or have risk-reducing gynaecological surgery to prevent endometrial and ovarian cancer [45]. The decision aid template was designed to be easily adapted for carriers of variants in other cancer predispositions genes such as *BRCA1* and *BRCA2* genes [33].

The aim of this paper is to present a case study and evaluation of PPIE in the Lynch Choices™ codesign project. Quantitative evaluation was performed using the PEIRS-22 validated survey, allowing comparison with other studies that had used this evaluation tool. Personal reflections from patients and public partners and a researcher are included to explore their perspectives. Complementary quantitative and qualitative data are presented to reflect on what went well, what could have gone better and to guide strategies to improve future research partnerships. This study adds to the evidence base regarding best practices for facilitating PPIE in research and presents recommendations for improvements in future.

## Methods

The Patient Engagement In Research Scale (PEIRS) was used, as a validated tool to enable systematic evaluation regarding the meaningfulness of PPIE in research and facilitate comparison with other studies. The shortened version called PEIRS-22 was chosen for brevity and convenience. Minor modifications were made to question wording to refer to the project being evaluated. Patients and public partners were invited to include optional free-text personal narratives, providing richer and more nuanced descriptions of their experiences. Researchers were invited to reflections to provide transparency about positionality and influence of the researcher’s background and viewpoints on the conduct, analysis and reporting of the study.

Selected demographic and three brief health literacy questions (adapted from [46]) were added to aid interpretation of responses in the context of people’s social context and acknowledge limitations regarding diversity of characteristics. The three screening questions are not powered to detect marginal health literacy but were brief enough to be included without making the survey overly long or intrusive.

Patient and public coauthors agreed that anonymity was not required. This was because of the small number of participants and the close relationships that had formed. However, some details were redacted to protect confidentiality through aggregating demographic characteristics and removing names from the individual PEIRS-22 scores and personal narratives.

In addition to the Patient Reference Panel from the CanGene-CanVar project (*n* = 12), key public partners from Lynch Syndrome UK (*n* = 1) and Lynch Syndrome Ireland (*n* = 1) were invited. The Invitation Letter, Participant Information Sheet, Consent Form and adapted PEIRS-22 (Supplementary File) were sent in an email from KK, with reminders after one, two and four weeks. Participants could return the documents as attachments or by pasting responses directly in an email. They were offered remuneration for their time, in line with national guidance [1].

The PEIRS-22 responses were collated by KK into an Excel spreadsheet. Following guidance from the PEIRS-22 developer (personal communication, KK and Dr Clayton Hamilton), responses to the 5-point Likert scale were converted to a numerical score: 4 for strongly agree, 3 for agree, 2 for neutral, 1 for disagree and 0 for strongly disagree. PEIRS-22 total scores were calculated by taking the total raw score for each participant across the 22 questions, dividing by 88 and then multiplying by 100. Total quantitative scores were calculated, along with the mean, median and standard deviation for each participant and the total group, for each question and overall. Qualitative data from free-text comments were reviewed via content analysis by KK to explore the latent meaning and context behind the words and present interpretive insights [47]. A mostly deductive approach to coding was applied, using the seven domains from the PEIRS framework as overarching themes. Some inductive coding was included to allow for the construction of subthemes to provide further reflections that were not captured by the predetermined framework domains. The positionality of the researcher (KK) is acknowledged, as a female who identifies as having White ancestry, in a position of power as a leading healthcare professional, with a desire to encourage partnerships between patient and public partners to improve understanding of personal priorities and improve health outcomes.

Results of the analysis were presented to all patient and public partners (included as coauthors) for discussion and revision before finalisation. Reporting was guided by the Guidance for Reporting Patient and Public involvement (GRIPP2) long-form checklist (Supplementary File) [16].

### Ethical approval

The ethics application and study documents were prepared by KK and presented to the research team for comments before submission. The study was approved by the Faculty of Environmental and Life Sciences Research Ethics Committee at the University of Southampton (reference number 100489).

## Results

Ten out of 12 (83%) Patient Reference Panel members, one Trustee of Lynch Syndrome UK and one Trustee of Lynch Syndrome Ireland, provided PEIRS-22 responses and wrote a personal narrative summarising their experience (overall response rate 12/14, 86%). Five participants were known to be Lynch syndrome carriers (from previous interactions). Self-reported demographic characteristics are presented in Table 1. Overall, nine identified as female (75%), one as genderqueer (8%) and two as male (17%). Most were in the age range of 41–60 years (*n* = 6/12, 50%) or 61–70 years (*n* = 5/12, 42%) and identified as having White British (*n* = 9/12, 75%) or any other White (*n* = 2/12, 17%) ancestry. All but two people had a previous cancer diagnosis, most commonly colorectal (*n* = 3), breast (*n* = 3) or endometrial (*n* = 2) cancer. Two people reported being diagnosed with two different primary cancers.

**Table 1:** Baseline demographic characteristics of survey respondents, by self-report

Baseline demographics	Responses (*n* = 12)	
	*n*	%
**Gender identity**		
Female	9	75
Genderqueer (she/they)	1	8
Male	2	17
**Age range (years)**		
18 to 25	1	8
41 to 60	6	50
61 to 70	5	42
**Ethnic group**		
White British	9	75
Any other White	2	17
Not specified	1	8
**Cancer diagnosis? (** ***n*** ** = 2 had two primary cancers)**		
Colorectal	3	25
Breast	3	25
Endometrial	2	17
Lymphoma	2	17
Skin (basal cell carcinoma)	1	8
Thyroid	1	8
None	2	17

Responses to the three brief health literacy screening questions are displayed in Table 2. Overall, the responses suggested that health literacy levels were high. Results indicated that there were no people with inadequate health literacy. The screening questions are not designed to assess marginal health literacy [46].

**Table 2:** Health literacy characteristics of survey respondents, by self-report. These were assessed via three short questions adapted from Chew et al. (2004) as a brief screening test for inadequate health literacy

Health literacy assessment question	Responses (*n* = 12)	
	*n*	%
**How often does someone (like a family member**,** friend**,** hospital/clinic worker**,** or caregiver) help you read hospital materials?**		
Never	10	83
Occasionally	1	8
Sometimes	1	8
**How confident are you filling out medical forms by yourself?**		
Extremely	8	67
Quite a bit	3	25
Somewhat	1	8
**How often do you have problems because you find it difficult to understand written information about medical conditions?**		
Never	1	8
Occasionally	10	83
Sometimes	1	8

**Fig. 1:**
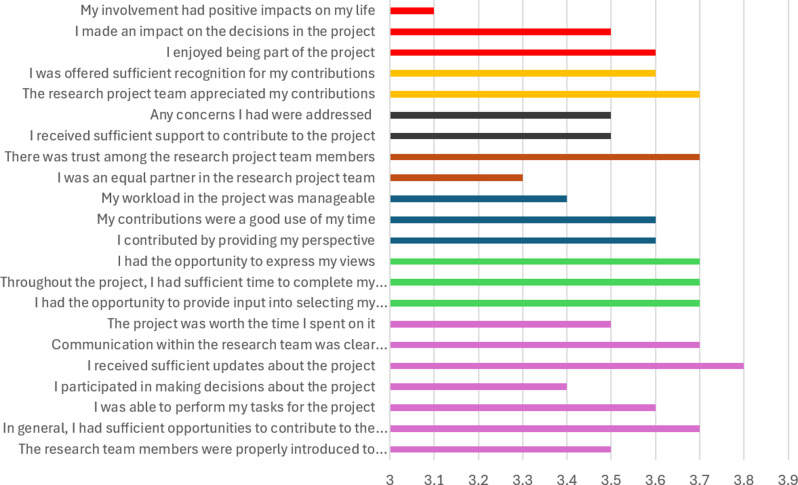
Mean scores for each PEIRS-22 question (*n* = 12 respondents)

All 12 people completed all 22 survey questions without asking for clarification from the researcher. The mean scores for each PEIRS-22 question are displayed in Fig. 1. The questions were spread across the seven PEIRS domains of meaningful PPIE in research: procedural requirements (PR), convenience (CN), contributions (CT), team environment and interaction (T), support (SU), feeling valued (FV) and benefits (BE). Overall, scores were high across all the domains, with mean scores all 3 or higher (corresponding to agree/strongly agree). There was no one domain where the scores were appreciably lower than those of the other domains.

**Key**:


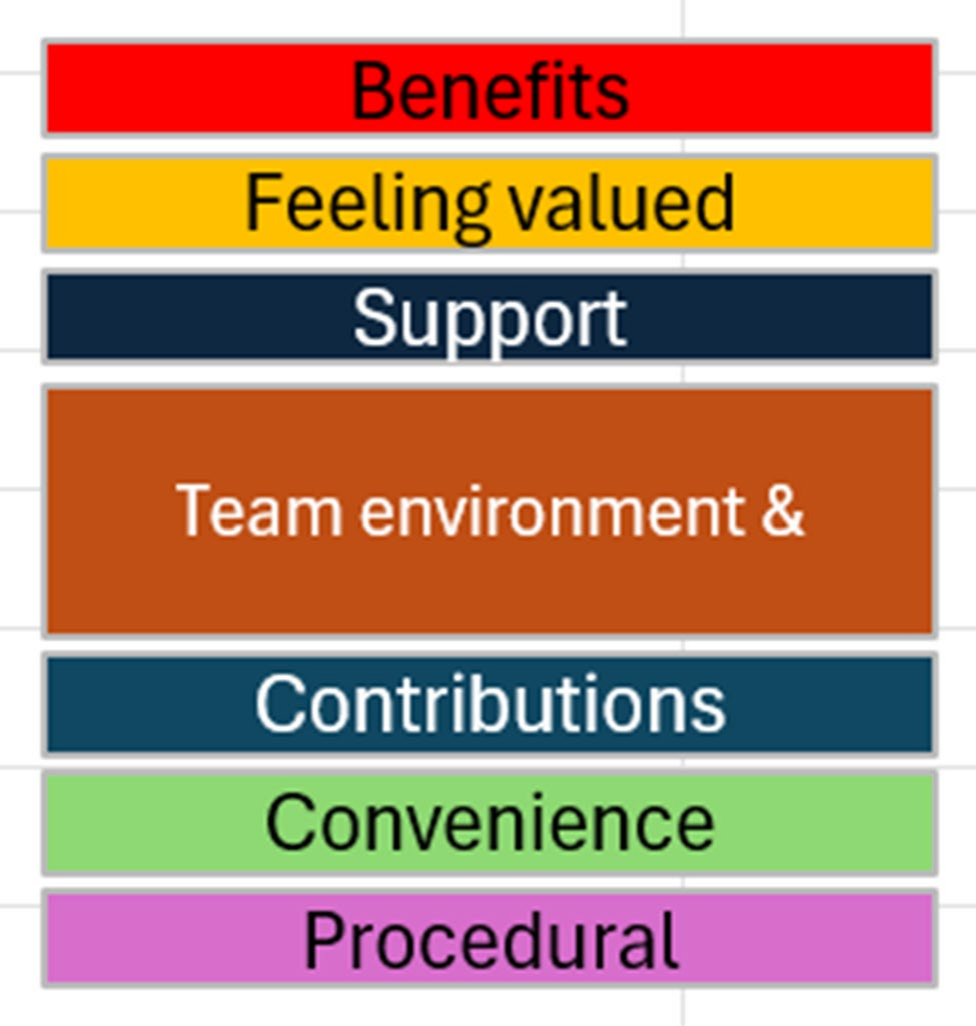


**Fig. 2:**
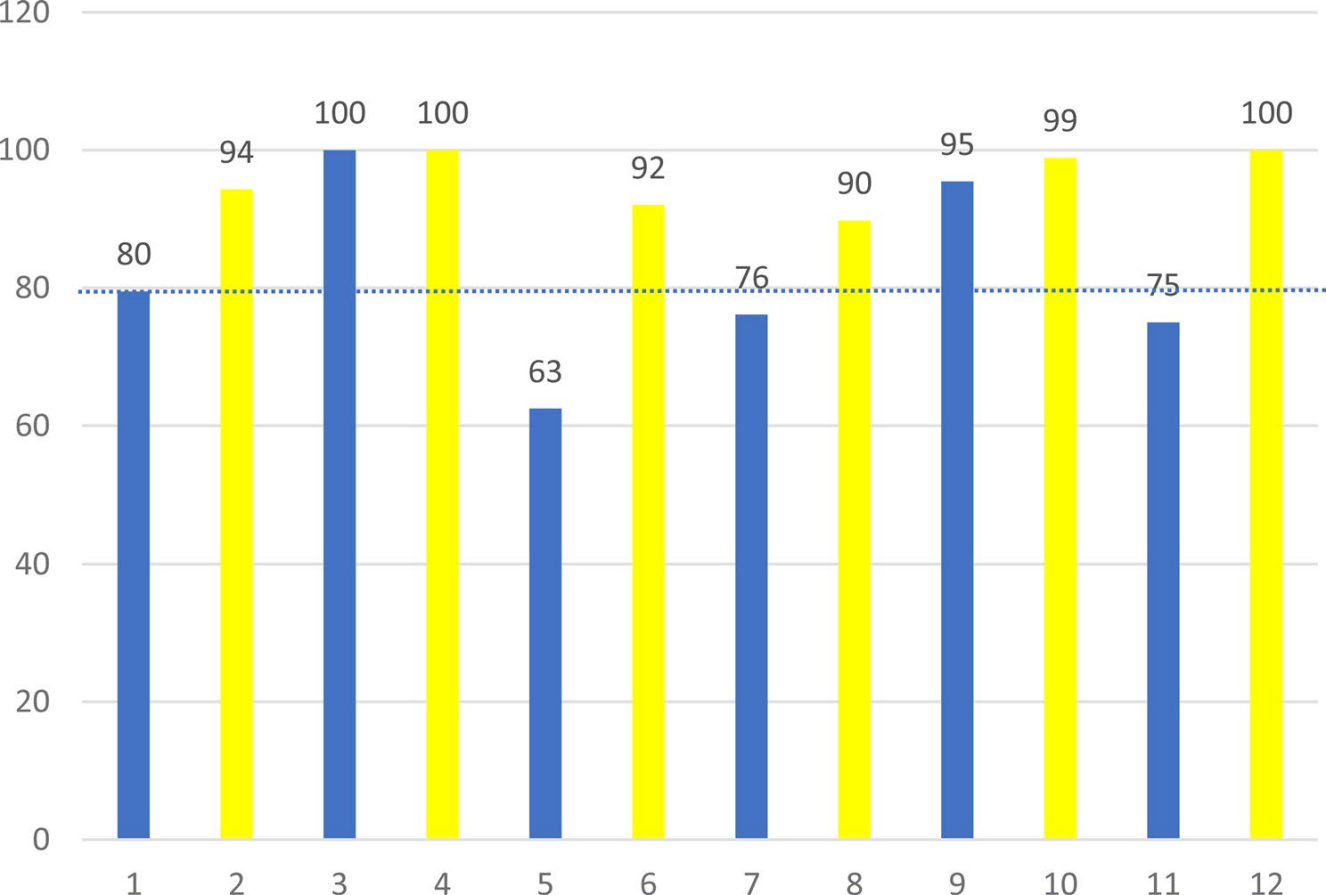
PEIRS-22 total scores for respondents. Note: The horizontal dotted line marks the threshold of 92 out of 100, with scores above this line interpreted as representing very meaningful engagement

The total PEIRS-22 scores for each person are displayed in Fig. 2. Across all responses, the total mean PEIRS-22 score was 89/100 (SD 12.4). The total median score was 93/100, which was above the threshold of 92, representing very meaningful engagement. The total scores were mostly high across all the domains, with 7/12 (84%) mean scores falling above the threshold for very meaningful engagement. However, it is important to note that not all scores achieved this level, suggesting room for improvement.

All respondents opted to include a free-text personal narrative to summarise their experience. All researchers (KK, KM, RF, KS, HH, DE, CF) were invited to include reflections, but there was only one response (KK) which limits the comprehensiveness of this narrative. The free text narratives are displayed in the Supplementary File.

Exemplar quote excerpts from the personal narratives were mapped to the seven themes in the PEIRS framework using deductive coding. These are displayed in Table 3. Subthemes were generated via inductive coding by KK via content analysis of personal narratives. This qualitative analysis was included to construct additional meanings that may not have been fully described by the PEIRS domains. For domain 1, Procedural Requirements, partners noted the importance of clear and regular communication, with transparency about the research aims: ‘The goal of the project was clear from the beginning’ (participant (p)12). With respect to domain 2, convenience, there were few relevant comments that may have indicated satisfaction with project requirements and the time allocated for tasks. The partners expressed motivation to share their unique views, with partnership in the project being a convenient way to achieve this: ‘After my experiences…I wanted to help in some way. I joined the Patient Reference Panel as part of this’ (p9). The third domain, Contributions, attracted many comments. The partners expressed being altruistic and motivated to make things better for others in the future: ‘Most of all, for me, I feel like in some small way if I can help one person on their journey, every minute is worthwhile’ (p3). Domain 4, Team Environment and Interactions, was also one of the main themes to which comments were mapped. Equal partnerships and two-way learning were experienced as ‘encouraging’ (p1), made one partner feel ‘proud’ (p10) and was viewed by another as ‘powerful’ (p11). Another commented that ‘the researchers were very open to input from the Patient Panel’ (p8). Support, the fifth domain, did not have as many comments mapped to it, perhaps due to a lack of concern that more support was needed. There were some positive reflections on the support that came from the relationships built during the project: ‘It made me feel like I wasn’t alone’ (p3), and there was a ‘spirit of comradery *[sic]*’ (p12). Feeling valued, the sixth domain, was a key theme. The partners expressed feeling that their contributions made a difference and were valued in the sixth domain: ‘Our opinions were valued and acted upon which is not always the case’ (p4), ‘Not only was my lived experience considered, it was respected’ (p6), ‘I have really valued the sense of voice and agency’ (p9), and ‘I felt respected and valued throughout the process – our ideas weren’t just heard, they genuinely informed decisions’ (p11). Finally, the seventh domain, Benefits, highlighted the time needed to build meaningful relationships and create impact: ‘Over the past five years, working with the research team has been incredibly rewarding’ (p2). This could have profound personal benefit: ‘I feel this is something that is such a healing process’ (p10). The experience could also bring feelings of helping the greater good: ‘encouraged by… the impact that this will make on the treatment and prevention of cancer’ (p1), ‘helping to shape Lynch choices, a highly valuable resource’ (p2). Involvement as a partner was perceived as ‘meaningful and purposeful’ (p10) and ‘truly rewarding’ (p11).

**Table 3:** Themes derived from PEIRS framework domains, subthemes and exemplar quotes from partners’ personal narratives

Themes corresponding to PEIRS domains (deductive) PEIRS-22 domain questions listed for reference	Subthemes (inductive)	Exemplar quotes from patient and public partner personal narratives
PROCEDURAL REQUIREMENTS (PR) PR2. The research team members were properly introduced to each other; PR9. In general, I had sufficient opportunities to contribute to the project; PR10. I was able to perform my tasks for the project; PR11. I participated in making decisions about the project; PR12. I received sufficient updates about the project; PR13. Communication within the research team was clear throughout the project; PR14. The project was worth the time I spent on it	Importance of clear and regular communciation; Transparency about research aims; Shared belief in the importance of the research	Although some of the medical terminology and use of acronyms could be confusing in some of the presentations, I have learnt a great deal from participating in the project (p1) I’ve had the opportunity to contribute in various ways—attending in-person meetings, presenting, and collaborating on the design and facilitation of focus groups (p2) I have been involved with patient advocacy for over 15 years, and I can honestly say that working with the CanGene CanVar research team was a privilege and an absolute pleasure. As a patient group we were kept informed and listened to throughout. (p4) The goal of the project was clear at the beginning. This gave a clear focus to what needed to be achieved. (p8) It’s great to have such comprehensive information across the many relevant LS topics available on a single platform that’s intuitive to individual needs. (p12)
CONVENIENCE (CN) CN1. I had the opportunity to provide input into selecting my tasks for the project; C3. Throughout the project, I had sufficient time to complete my tasks for the project; CN4. I had the opportunity to express my views	Motivation to share views from unique experiences	After my experiences with genetic counselling, I decided I wanted to help in some way. I joined the Patient Reference Panel as part of this. I was keen to, as far as I could, try to ensure that someone else in my position wouldn’t be told the same, or something similar. I feel that the way in which we present choices to patients, and give them the information to make informed decisions, is so important. (p9) Even though my specific diagnosis wasn’t linked to inherited cancer risk, I understood the burden and uncertainty patients carry — and the need for clear, compassionate, co-designed tools to support their choices. (p10)
CONTRIBUTIONS (CT) CT1. I contributed by providing my perspective; CT2. My contributions were a good use of my time; CT4 My workload in the project was manageable	Altruism; Desire to improve care	My own experience with cancer, along with the support I received through and beyond genetic testing, motivated me to join the CanGene-CanVar programme, hoping to make a difference for others in similar situations (p2) Most of all, for me, I feel like in some small way if I can help one person on their journey, every minute is worthwhile. (p3) It is so long since I made a contribution to the project that I have answered ‘Neutral’ to many questions. However, it has been such a well-run project from the PPI perspective that I didn’t feel I was needed! (p5) I am incredibly proud of being a patient rep for CanGene-CanVar, everything it has achieved and how it has involved patients in the achievement of it. It has been a fantastic opportunity to get patient voice into decision making and ensuring that decisions included consideration of the impact on patients and their needs. (p6) [My motivation to take part was experiencing] limited support and information for my family (including young son) when I was subject to bowel cancer, surgery and chemo (over 10 years ago). My goal was to help change this for others experiencing the same life journey. Happy to help. (p7) I did not have good experiences with genetic counselling. I was only 21, and they kept reminding me of that. I knew I wanted to have surgery, as I had watched my Dad go through cancer treatment and I wanted to reduce my own risk as much as possible… I was borderline reprimanded and told I was “too young to be making those kinds of decisions”. What they didn’t account for was that dealing with a close family member with cancer and a BRCA2 diagnosis forces you to grow up. It was clear that they saw my age on a form or piece of paper, rather than viewing me as a whole person. I had a bilateral risk-reducing mastectomy with immediate reconstruction, when I was 23 years old. So far in my life, it is the best decision I have ever made. (p9) Although I don’t have Lynch Syndrome or a known BRCA gene, I have lived through breast cancer… I underwent aggressive treatment… [that] later led to endometrial hyperplasia, which meant I had to have a full hysterectomy and bilateral oophorectomy – all before the age of 50, while raising a young family and caring for elderly parents. This personal journey made me deeply aware of the emotional, physical, and practical complexities of making cancer-related decisions. It’s why I was so committed to the CanGene-CanVar decision aid project. (p10) It gave me the chance to contribute lived insights and perspectives that shaped something meaningful and tailored to the needs of real people and families (p11) As a patient advocate for people who are affected by Lynch Syndrome in Ireland, being on the ground with families as they navigate their diagnosis and seeing the repeated difficulties faced in obtaining timely information and supports needed to aid complex decision making when contending with fragmented pathways & services, and conflicting information, having the opportunity to contribute to this body of work - CanGene-CanVar decision aid codesign project, the creation of an easily accessible, trustworthy and practical aid that meets the information and support requirements of all who use it, both patients and clinicians alike, was like winning the lotto to be honest. (p12)
TEAM ENVIRONMENT AND INTERACTIONS (T) T2. I was an equal partner in the research project team; T5. There was trust among the research project team members	Two-way learning	.[I was] encouraged by the amount of work and enthusiasm shown by those involved in the research. (p1) The work carried out is a rare example of true co-design. As patients we felt useful and an integral part of the team. (p4) I… had the opportunity to learn from the academics we worked with and their research findings as well as meet some brilliant people, including my co patient reps who I hope to continue to work with in the future. (p6) The researchers were very open to input from the Patient Panel. With a range of different patient representatives it wasn’t possible to incorporate all views as sometimes they were not aligned. (p8) I was proud to be treated as an equal partner throughout. (p10) It’s been powerful to see how collaboration between professionals and those with lived experience can create something so much more impactful and human. (p11) From first introductions, to me writing this, and everything in-between, it’s been the most rewarding and I daresay fun experience. The genuine dedication of the research team to truly listen to and understand the experiences and needs of people living with Lynch and to work together to co- design and develop a resource that would help lessen their burdens has been truly heartwarming. (p12) Kelly and the research team have been incredibly supportive, empowering and professional throughout, treating me as a valued team member whose contributions are important. It was wonderful to see my thoughts and suggestions shape the project from beginning to end- this wasn’t a box ticking exercise; this was meaningful partnership. We made a great team and patients will benefit from what we’ve all achieved by working together. I’m extremely grateful to Kelly and every person involved in this project for everything they do to improve the lives of people living with Lynch syndrome. The whole affair was a masterclass in PPI - empathy, inclusion and science communication at its finest, and a pleasure to be part of. (p12)
SUPPORT (SU) SU1. I received sufficient support to contribute to the project; SU2. Any concerns I had were addressed	Importance of building relationships	The whole panel were so nice, they all listened to each person’s story, each person’s opinions and ideas and I think I speak for everyone in that we were all made to feel like we mattered and that there was a way that we could help other people that may find themselves in the same situation. It made me feel like I wasn’t alone, I didn’t have much confidence at first but I was always encouraged to speak and to engage in discussions. I have been given opportunities to publicly speak, to write blog posts and to also record a podcast. I even spent a week with the ethics group and travelled to [a conference]. (p3) The researchers spent time explaining what changes they had implemented and why in various drafts as we went through the iterative process of design. (p8) The level of involvement and communication throughout, so brilliantly guided by Kelly, created a culture of trust, respect and comradery. (p12)
FEELING VALUED (FV) FV1. The research project team appreciated my contributions; FV3. I was offered sufficient recognition for my contributions	Listening to and respecting experiences; Feeling that contributions made a difference	I felt honoured to be part of the project (p1) [I] felt that I was listened to and valued during the discussions (p1) Being part of the CanGene-CanVar PPI team has been one of the best projects that I have taken part in. I joined the team after my own cancer journey and subsequent genetic diagnosis of Lynch Syndrome. Everybody has their own story and each is as important as the next and this is exactly the way I was made to feel as part of the PPI panel. (p3) Our opinions were valued and acted upon which is not always the case. (p4) Not only was my lived experience considered, it was respected. (p6) I was made to feel valued, not just by being offered compensation but by being offered the opportunity to be a co-author on papers. For me the latter is more important. (p8) I have really valued the sense of voice and agency that the PRP has given me. (p9) I felt respected and valued throughout the process—our ideas weren’t just heard, they genuinely informed decisions. (p11)
BENEFITS (BE) BE1. I enjoyed being part of the project; BE2. I made an impact on the decisions in the project; BE4. My involvement had positive impacts on my life	Time needed to develop impact and build relationships	…encouraged by…the impact that this will make on the treatment and prevention of cancer. (p1) Over the past five years, working with the research team has been incredibly rewarding. (p2) I’ve also really enjoyed networking with others and helping to shape Lynch Choices, a highly valuable resource for people diagnosed with Lynch syndrome. (p2) I feel this is something that is such a healing process. I still have a way to go in that regard but I do feel much more confident than I was. I will definitely be sad to see the end of this project but I have come away with so much knowledge and made some epic friends. (p3) Being involved as a public contributor felt meaningful and purposeful. (p10) Being a partner in the co-design of Lynch Choices has been a truly rewarding experience. (p11)

## Discussion

PEIRS-22 scores and personal narratives indicate mostly positive experiences with PPIE in the Lynch Choices™ codesign project, with some suggestions for future improvements. The median score (93/100) exceeded the threshold for ‘very meaningful engagement’ [5] and qualitative feedback highlighted trust, clear communication, and feeling valued as key drivers of positive experiences. Participants described their involvement as rewarding and purposeful, with perceived personal benefits and confidence that their contributions influenced project outcomes.

Codesign was a guiding principle for the development of Lynch Choices™, prioritising PPIE and in-depth research to inform the design of an intervention that was flexible and tailored to the individual context [39, 48–50]. The PEIRS-22 results from the Patient Reference Panel suggested high satisfaction with the relationships established during the project (domain T, Team Environment and Interactions). Panel members mostly felt that their views had an impact on decisions (domain BE, benefits). While the Patient Reference Panel largely felt they were able to participate in decision-making (domain PR, Project Requirements), this was a group with high literacy and most shared characteristics, such as White British ethnicity, similar age and experience with cancer diagnoses. A group with more heterogeneous characteristics may have had different views, interactions and experiences.

A larger PEIRS-22 study of international PPIE participants in the OMERACT project reported positive experiences, though with a lower response rate (40%, 68/172) and a median score of 73/100, above the meaningfulness threshold across all subdomains [51]. Lower scores correlated with lower self-reported engagement, and nonresponders may have had less positive experiences. When asked about the scale’s comprehensiveness, 20% felt neutral and 18% disagreed or strongly disagreed, highlighting the value of adding self-rated engagement and free-text comments alongside Likert ratings [51]. Including a similar question in the Lynch Choices™ study could have enabled comparison with PEIRS-22 responses.

Other studies using PEIRS-22 have reported similar positive experiences. The Cochrane Review team found high post-project satisfaction, with 76% extremely satisfied and 11% very satisfied, and qualitative comments reflected enjoyment of coproduction despite challenges around resources and tailoring responsibilities [30]. Arola et al. (2025) explored experiences of cocreation among older adults, identifying three key themes: diversity to enrich understanding, interactive activities to create knowledge, and supportive environments to enhance collaboration [52].

Christiansen et al. (2023) translated and validated a Danish version of PEIRS-22 with cancer advisory board members, reporting a high overall score (85/100), indicating effective PPIE integration [53]. Supplementing survey data with focus groups revealed the importance of trust-building and clarified motivations for involvement. This mixed-methods approach offers deeper insights than quantitative data alone and aligns with our inclusion of free-text comments and personal narratives. Future studies could further enhance understanding through interviews or focus groups.

Wong et al. (2024) explored repeated use of PEIRS-22 in dementia research through reflexive thematic analysis, finding the tool broadly useful for tracking engagement but burdensome for some participants, particularly those with early-stage dementia [54]. These findings highlight the need to consider personal needs and social context early in projects to set realistic expectations and allow flexibility.

To ensure transparency and flexibility, the Lynch Choices™ team agreed Terms of Reference with partners, emphasizing voluntary participation [32]. The PEIRS-22 evaluation was treated as an optional activity, achieving a high response rate after reminders. While useful for comparison, the formal survey felt less aligned with other PPIE interactions. A pre-study assessment could have clarified expectations, and future work should consider adapting PEIRS-22 for greater engagement. Wong et al. (2024) found that adding comment boxes and emojis improved personalization and feedback quality [54].

Some panel members were Lynch syndrome carriers (*n* = 5) but did not represent all carriers, such as younger individuals unaffected by cancer. To broaden perspectives, partnerships included Lynch Syndrome UK, Lynch Syndrome Ireland, and other charities. Similar collaborations were used successfully by Morel et al. (2023) to leverage trust and connections within patient communities [55].

Ideally, codesign should include views from all intended users to ensure relevance and cultural sensitivity, but resource constraints often make this impractical. Consequently, patient advocates and trustees frequently represent wider communities, and some become national or international experts [3]. Examples from our coauthors include roles at major conferences: Cancer Research UK Early Detection (HW, 10/2023), Canadian Association of Psychosocial Oncology (LT, 06/2023, see blog post CAPO Conference 2023: A Co-design Workshop) and European Hereditary Tumour Group (JY, 09/2023, see blog post The Co-design of Patient Information Leaflets – A Patient’s Experience). Trustees of Lynch Syndrome UK and Lynch Syndrome Ireland have led engagement through hybrid conferences, websites, email groups, and social media, encouraging diverse input, though some members may still feel unable to speak up.

Accessible education about research and PPIE can help diversify patient involvement. For example, the Independent Cancer Patient Voice charity offers study days and a five-day course for advocates (see https://www.independentcancerpatientsvoice.org.uk/). While valuable, such opportunities may be inaccessible for people with low income, caring responsibilities, or disabilities. Researchers can also use training and frameworks to improve readiness for PPIE [12, 15, 31, 56]. Inclusion of marginalized groups requires community partnerships to build trust [57] and dedicated funding to avoid widening health inequalities. The Lynch Choices™ team secured additional funding to co-create decision aid images with charities serving marginalised populations [58]. There is no one-size-fits-all approach to inclusivity. Future work should explore varied views and preferences.

Free-text comments and personal narratives highlighted the mostly positive impact of involvement for patients, public partners, and researchers, including feeling valued and contributing meaningfully. Some described altruistic motivations to help others and improve patient information and decision support. Partners emphasised respect, honest two-way communication, and clear evidence that their input influenced outcomes. Relationships strengthened over the six-year project, supported by fully costed PPIE and regular meetings, which may have contributed to higher engagement scores. However, responses at earlier timepoints might have differed. Barn et al. (2022) similarly noted the importance of investing time and energy to foster respect and value within teams, especially when in-person interactions are limited [59].

High PEIRS-22 scores and mostly positive narratives indicate that the Lynch Choices™ codesign team delivered valued PPIE experiences. The study PPIE was well costed, but there was still room for improvement regarding more funding for activities, time for communication and swifter payment mechanisms. This case study shows that PPIE can meaningfully shape complex health interventions, benefiting patients, public partners, and researchers while fostering future collaboration. Placing patient priorities at the centre of research is essential for improving health outcomes, though challenging amid limited resources. Funders should prioritise dedicated PPIE budgets, long-term support, and flexibility in funding models to enable inclusive, codesigned research. With clear shared aims and investment in trusting relationships, the benefits from two-way learning are innumerable.

### Strengths

PPIE from conception of the idea throughout the lifecycle of the research project was a strength. This helped foster engagement and understanding between researchers, patient and public partners. Learning was two-way, benefitting both groups. A bonus of the partnership was the close relationships, peer support and encouragement experienced by the people involved. This not only strengthened satisfaction and willingness to partner in future research but also had a cascade effect of encouraging other patients to consider PPIE (personal communication between KK and partners). During codesign of the decision aid, many suggestions from PPIE helped to optimise the decision aid before it was launched in clinical practice. Active involvement in dissemination fostered awareness and trust among the partners’ networks, which expanded the number of people who viewed and interacted with Lynch Choices™.

### Limitations

Given the closeness of relationships, it may have been preferable for an independent researcher to have conducted the evaluation. However, there was no one available and no funding for this. This was a paper about the patient and public voice, however there was limited uptake from patient and public partners to write all or part of the manuscript. All preferred that the researcher prepare the first draft for review. All agreed to meet the coauthorship requirements. However, only a few PPIE contributors provided suggestions such as changes to wording and adding or deleting sentences in the body of the manuscript. Several did take an active role in revising and rewriting the Plain English Summary. Personal narratives were also written in PPIE contributors’ own words, with excerpts presented in the paper verbatim. Only one researcher provided a narrative, limiting the contribution from the researcher perspective.

The risk of social desirability bias [60] is acknowledged, which could have skewed results toward a more favourable outcome because partners presented views that were perceived to be more socially acceptable than their true feelings. The researcher attempted to minimise this by encouraging any feedback, including what went well and what did not go as well. Anonymity was considered, but due to the small, close group and lack of an external researcher to collate the data, it was unanimously agreed that anonymity was not needed or possible. The relationships built during the project may have also minimised social desirability bias because feedback about decision aid development was often critical, especially in the early stages. This demonstrated that people felt safe and respected enough to share critical views. The feedback was recorded in a Table of Changes [39] and transparently used where appropriate to improve the prototype before proceeding to the next stages of research.

The one-off PEIRS-22 was distributed near the end of this research project. In the future, a pre-project assessment could also be considered to facilitate systematic clarification of goals and expectations and allow tracking of any changes related to engagement, expectations and experiences over time. The sample size was small because of the level of commitment required by Patient Reference Panel members and public partners to be part of the core research team. Since they did not respond to the email invitation or reminders, it is unclear whether the two panel members who did not participate had fewer positive experiences, were unavailable, or chose not to take part. It was also noted that they had not engaged in activities during the later stages of the research project. It had been some time since at least some participants had been actively involved in study activities, which may have introduced some recall bias.

There was some variation in health literacy levels, but overall, there was a lack of representation from marginalised communities or participants with inadequate health literacy. Effort is needed to involve these groups in future research, with appropriate support and resources to recruit participants in diverse communities, offer flexible participation opportunities that are engaging and relevant. These strategies are needing to learn from the experiences of underserved groups.

## Conclusions

This evaluation of PPIE in research via quantitative PEIRS-22 data and qualitative personal narrative data provided a rich description of the two-way communication and trusted relationships between researchers, patients and public partners in the Lynch Choices™ codesign project. The total mean PEIRS-22 score was high (93/100), with key reasons for this explained in the personal narratives. The mostly positive feedback seemed to be driven by feelings of value and team interactions. Trusted relationships between patients and public partners and researchers took time to build, which should be factored into early project planning and costings. Most partners believed that being part of the team was personally beneficial and that the project outputs would help others. This study adds to the body of evidence regarding experiences with PPIE in research. Reflections and encouragement are provided for future researchers considering a similar approach.

## Supplementary Information

Below is the link to the electronic supplementary material.

Supplementary Material 1

## Data Availability

The datasets used and analysed during the current study are available from the corresponding author upon reasonable request.

## Abbreviations

GRIPP2: Guidelines for Reporting Patient and Public Involvement
PEIRS-22: Patient Engagement in Research Scale (22-item version)
PPIE: Patient and Public Involvement and Engagement
