# Supplementary material for: Experiences of patients and public partners in codesign of Lynch Choices™: an evaluation study using the Patient Engagement In Research Scale (PEIRS-22)
Source: Res Involv Engagem. 2026 Mar 9;12:46. doi: 10.1186/s40900-026-00854-z (PMC13085504; doi:10.1186/s40900-026-00854-z)
Supplement: Supplementary file 1 — Supplementary Material 1 [file 40900_2026_854_MOESM1_ESM.docx]

**Supplementary File**

**Title: Experiences of patients and public partners in codesign of Lynch Choices^TM^: An evaluation study using the Patient Engagement in Research Scale (PEIRS-22)**

**Authors:** Kelly Kohut*^1,2,3^, Lesley Turner^4^, Caroline Dale^4^, Sue Duncombe^4^, Rochelle Gold^4^, Sonia Patton^4^, Richard Stephens^4^, Frankie Vale^4^, Helen White^4^, Steve Worrall^4^, Julie Young^4^, Tracy Smith^5^, Roberta Horgan^6^, Kate Morton^2^, Becky Foster^2^, on behalf of the CanGene-CanVar Patient Reference Panel, on behalf of the International Lynch Decision Aid Expert Partner Panel, Diana Eccles^7^, Claire Foster^2^

^1^ Department of Clinical and Biomedical Sciences, University of Exeter Medical School, Exeter, UK
^2^ Centre for Psychosocial Research in Cancer: CentRIC, School of Health Sciences, University of Southampton, Southampton, UK
^3^ Centre for Genomic Medicine, St. George’s University Hospitals NHS Foundation Trust, London, UK
^4^ CanGene-CanVar Patient Reference Panel, Southampton, UK
^5^ Public contributor, Lynch Syndrome UK
^6^ Public contributor, Lynch Syndrome Ireland
^7^ Faculty of Medicine, University of Southampton, Southampton, UK

***Correspondence:**
Kelly Kohut, [k.e.kohut@exeter.ac.uk](mailto:k.e.kohut@exeter.ac.uk); [kelly.kohut@stgeorges.nhs.uk](mailto:kelly.kohut@stgeorges.nhs.uk)
**ORCID:** 0000-0002-9852-2872

Contents

[Invitation 2](#_Toc206423880)

[Participant information sheet 3](#_Toc206423881)

[Consent form 6](#_Toc206423882)

[Survey incorporating Patient Engagement in Research Scale (PEIRS-22) 8](#_Toc206423883)

[GRIPP2 (guidance for reporting patient and public involvement) long-form checklist 17](#_Toc206423884)

[Personal narratives 25](#_Toc206423885)

# Invitation

**Draft email invitation to participants**

**Study Title:** Patient Engagement in Research Scale (PEIRS) assessment of the quality of patient engagement in the CanGene-CanVar decision aid codesign project

**Researcher:** Kelly Kohut

**University email:** [redacted]

**Ethics/ERGO no:** 100489

**Version and date:** version 1, 27/12/2024

I am inviting you to participate in a study regarding your experience as a patient panel member or patient and public partner with the CanGene-CanVar Lynch Choices decision aid codesign project. The aims of this research study are to gain knowledge by directly asking you about your experience being involved with the project. A survey called PEIRS (Patient Engagement in Research Scale) will be used to ask your opinions about the quality of patient engagement in the project. This will help the research team to understand what went well and what could be done better in future research projects.

This study was approved by the Faculty Research Ethics Committee (FREC) at the University of Southampton (Ethics/ERGO Number: 100489).

Please take as much time as you need to review the attached documents:

-participant information sheet

-consent form

I will write to follow up in 1, 2 and 4 weeks to check if you are interested in participating. Please feel welcome to contact me with any questions in the meantime.

Once you have let me know, I can send you the survey by email to complete in your own time. You can send this back to me by email. If you have any issues, you will be welcome to contact me directly for assistance.

Thank you for your consideration.

Kind regards,

Kelly Kohut

PhD student, University of Southampton

# Participant information sheet

**Study Title:** Patient Engagement in Research Scale (PEIRS) assessment of the quality of patient engagement in the CanGene-CanVar decision aid codesign project

**Researcher:** Kelly Kohut

**University email:** [redacted]

**Ethics/ERGO no:** 100489

**Version and date:** version 2, 27/12/2024

**What is the research about?**

My name is Kelly Kohut and I am a PhD student in Health Sciences at the University of Southampton in the United Kingdom.

I am inviting you to participate in a study regarding your experience as a patient panel member or patient and public partner with the CanGene-CanVar decision aid codesign project. The aims of this research study are to gain knowledge by directly asking you about your experience being involved with the project. A survey called PEIRS (Patient Engagement in Research Scale) will be used to ask your opinions about the quality of patient engagement in the project. This will help the research team to understand what went well and what could be done better in future research projects.

This study was approved by the Faculty Research Ethics Committee (FREC) at the University of Southampton (Ethics/ERGO Number: 100489.

**What will happen to me if I take part?**

This study involves completing a questionnaire which should take approximately 15 minutes of your time. If you are happy to complete this survey, you will need to tick (check) the box below to show your consent. As you are known to the researcher and have been a partner in the research study, the researcher will know whether you have participated and what answers you provided. Demographic questions will be asked to collect information about participant characteristics such as age, gender identify and education in case this is relevant for interpreting the findings and acknowledging any limitations in terms of diversity of the group.

A separate open text question will be provided in a separate link for people to share their personal story in words, if they choose to do so. There will be the option of having your name attached to this story, if you would like. All patient and public partners are already known to the researcher over the past 4.5 years and they will be invited to be co-authors on the manuscript publishing the results of the survey. This is a personal choice.

**Why have I been asked to participate?**

You have been asked to take part because you have been a member of the CanGene-CanVar Patient Reference Panel (including up to 13 members from different parts of the UK) or a patient and public contributor from the Lynch Syndrome UK, Lynch Syndrome Ireland or South East London patient groups or charities. You have all been significantly involved in the project to codesign a patient decision aid to support decisions about genetic cancer risk management, called Lynch Choices.

I am aiming to recruit around 15 participants for this study.

**What information will be collected?**

A published, validated survey instrument called PEIRS-22 will be used. The questions in this survey ask for information in relation to the quality and experience of patient engagement in research from a patient partner perspective. The questions are regarding different aspects of project participation, including procedures, convenience, team environment and interaction, support and benefits. The questions include Likert-scale rations, asking participants to tick a box corresponding to strongly agree, agree, neutral, disagree or strongly disagree for each statement. The information is not expected to be distressing, upsetting or particularly sensitive. The participants already have a strong relationship and rapport with each other and the researcher from involvement in the project over the past 4.5 years. Links will be provided to contact the researcher before or after the survey for support and debriefing, if helpful. Links for NHS clinical services will be provided in case participation brings up questions or concerns related to participants’ health or care that cannot be addressed in the context of the research study.

Demographics including age range, gender identity, cancer history and personal history of a genetic cancer predisposition will be asked to provide context to the interpretation of the results.

The final survey question will contain a free text box where you will be asked to type in your personal story, if you wish. Please note that it is up to you whether you will be happy to have this quote published with your name. However, since you have been a key team member in this research and will be invited to be a named co-author on the manuscript, you will be invited to share your name if you would like to do so. This is optional. You can provide an anonymous story for the manuscript if you prefer. This will still be valued.

You do not have to answer all the questions if you do not wish to do so.

**What are the possible benefits of taking part?**

You will be offered remuneration for your time. You will be offered the choice to be a named co-author on the manuscript to be submitted for publication in a peer-reviewed journal. The study will also be submitted for publication at national or international meetings. It is possible you may receive additional offers to take part in other research projects based on this study, if you are interested. If you decide to take part in this study, you will not receive any other direct benefits; however, your participation will contribute to knowledge in this area of research.

**Are there any risks involved?**

It is expected that taking part in this study will not cause you any psychological discomfort and/or distress, however, should you feel uncomfortable you can leave the survey at any time or contact the following resources for support:

<https://www.nhs.uk/>

111.nhs.uk or call 111

For life-threatening emergencies: call 999

**What will happen to the information collected?**

All information collected for this study will be stored securely on a password protected computer and backed up on a secure server via the University of Southampton OneDrive/SharePoint system. Qualitative data will be collected via the open text box responses and shared with permission as patient stories in the manuscript and/or presentations about the study. This will only have people’s names attached if they decide to give permission for this. Only the researcher, their supervisors and research team will have access to the survey responses.

The information collected will be analysed and written up for submission to a scientific, peer-reviewed journal for consideration of publication. It may be shared in presentations at conferences, meetings and public engagement events.

The University of Southampton conducts research to the highest standards of ethics and research integrity. In accordance with our Research Data Management Policy, data will be held for 10 years after the study has finished when it will be securely destroyed.

**What happens if there is a problem?**

If you are unhappy about any aspect of this study and would like to make a formal complaint, you can contact the Head of Research Integrity and Governance, University of Southampton, on the following contact details: Email: [rgoinfo@soton.ac.uk](mailto:rgoinfo@soton.ac.uk), phone: + 44 2380 595058.

Please quote the Ethics/ERGO number above. Please note that by making a complaint you might be no longer anonymous.

More information on your rights as a study participant is available via this link:

<https://www.southampton.ac.uk/about/governance/participant-information.page>

**Thank you for reading this information sheet and considering taking part in this research.**

# Consent form

**Study Title:** Patient Engagement in Research Scale (PEIRS) assessment of the quality of patient engagement in the CanGene-CanVar decision aid codesign project

**Researcher:** Kelly Kohut

**University email:** [redacted]

**Ethics/ERGO no:** 100489

**Version and date:** version 2, 27/12/2024

Thank you for your interest in this study. It is very important to us to conduct our studies in line with ethics principles, and this Consent Form asks you to confirm if you agree to take part in the above study. Please carefully consider the statements below and add your initials and signature only if you agree to participate in this research and understand what this will mean for you.

**Please add your initials to the boxes below if you agree with the statements:**

| **Mandatory Consent Statements** | **Participant Initials** |
| --- | --- |
| I confirm that I read the Participant Information Sheet version v2, dated 27/12/2024 explaining the study above and I understand what is expected of me. |  |
| I was given the opportunity to consider the information, ask questions about the study, and all my questions have been answered to my satisfaction. |  |
| I agree to take part in this study and understand that data collected during this research project will be used for the purpose of this study. |  |
| I understand that my participation is voluntary and that I am free to withdraw from this study at any time without giving a reason. |  |

**Additional Statements - please add your initials in the boxes below you to agree to:**

| **Additional Consent Statements** | **Participant Initials** |
| --- | --- |
| I give permission for my personal story in the free text box section of the questionnaire to be published in a paper in a scientific journal and presented at conferences with my name attached. |  |
| I understand that my contact details will be retained by Kelly Kohut, [email redacted] and the research team at the University of Southampton so that I can be contacted about future research studies relevant to this project/research filed, for which I may be suitable. I understand that agreeing to be contacted does not oblige me to participate in any further studies. |  |
| I understand that my personal data will be kept for 10 years and that I can access my details or ask for them to be removed from the contact list at any time by contacting the research team at the University of Southampton. |  |

| ______________________________ | ________________________ | __________________ |
| --- | --- | --- |
| Name of participant | Signature | Date |
|  |  |  |
| _____________________________ | ________________________ | __________________ |
| Name of person taking consent | Signature | Date |

*Once this Consent Form has been signed by all parties, a copy of the signed and dated form should be provided to the study participant. Original signed copy should be stored in the study site file (If applicable).

# Survey incorporating Patient Engagement in Research Scale (PEIRS-22)

*Author’s note: The PEIRS-22 template was modified to add words referring to the decision aid codesign project. Demographic and health literacy assessment questions were added. An invitation to add a free text personal narrative was added at the end of the survey.*

**Study Title:** Patient Engagement in Research Scale (PEIRS) assessment of the quality of patient engagement in the CanGene-CanVar decision aid codesign project
**Researcher:** Kelly Kohut
**University email: [redacted]
Ethics/ERGO no:** 100489
**Version and date:** version 1, 12/11/2024

**SURVEY**

**Patient Engagement In Research Scale – PEIRS-22**

**INSTRUCTIONS:** Thinking about your experience as a patient and public partner involved the CanGene-CanVar decision aid codesign project, please respond to the statements by choosing only one box for each statement. If you are unsure about which option to choose for a statement, please give the best response you can. This survey may take you about 3 to 7 minutes to complete.

Demographic questions:

1. **How old are you?**

- 18 to 25
- 26 to 40
- 41 to 60
- 61 to 70
- Over 70

1. **What is your gender identity?**

Male

Female

Other/ Prefer to self-describe: (optional to add text):___________________

Prefer not to say

1. **What is your ethnic group?**

- Black Caribbean
- Black African
- Black Other
- Bangladeshi
- Chinese/ South East Asian
- Indian
- Pakistani
- White British
- Any other White
- Asian
- Other: __________________________________________________
- Prefer not to say

1. **Have you ever been diagnosed with cancer?**

- No
- Bowel (colon) cancer
- Endometrial (womb/uterus) cancer
- Other (please state here): ___________________________________

Finally, we have 3 short questions we would like to ask you about how easy you find it to understand health information (adapted from Chew et al. 2004).

1. **How often does someone (like a family member, friend, hospital/clinic worker, or caregiver) help you read hospital materials?**

(1) Always

(2) Often

(3) Sometimes

(4) Occasionally

(5) Never

______________________________________________________

1. **How confident are you filling out medical forms by yourself?**

(1) Extremely

(2) Quite a bit

(3) Somewhat

(4) A little bit

(5) Not at all

______________________________________________________

1. **How often do you have problems because you find it difficult to understand written information about medical conditions?**

(1) Always

(2) Often

(3) Sometimes

(4) Occasionally

**Survey questions:**

**Procedural Requirements**

The following seven (7) statements are about your general experiences throughout the project.

| PR2. | The research team members were properly introduced to each other | | | | |
| --- | --- | --- | --- | --- | --- |
|  | Strongly Agree  **☐** | Agree  **☐** | Neutral  **☐** | Disagree  **☐** | Strongly Disagree  **☐** |

| PR9. | In general, I had sufficient opportunities to contribute to the project | | | | |
| --- | --- | --- | --- | --- | --- |
|  | Strongly Agree  **☐** | Agree  **☐** | Neutral  **☐** | Disagree  **☐** | Strongly Disagree  **☐** |

| PR10. | I was able to perform my tasks for the project | | | | |
| --- | --- | --- | --- | --- | --- |
|  | Strongly Agree  **☐** | Agree  **☐** | Neutral  **☐** | Disagree  **☐** | Strongly Disagree  **☐** |

| PR11. | I participated in making decisions about the project | | | | |
| --- | --- | --- | --- | --- | --- |
|  | Strongly Agree  **☐** | Agree  **☐** | Neutral  **☐** | Disagree  **☐** | Strongly Disagree  **☐** |

| PR12. | I received sufficient updates about the project | | | | |
| --- | --- | --- | --- | --- | --- |
|  | Strongly Agree  **☐** | Agree  **☐** | Neutral  **☐** | Disagree  **☐** | Strongly Disagree  **☐** |

| PR13. | Communication within the research team was clear throughout the project | | | | |
| --- | --- | --- | --- | --- | --- |
|  | Strongly Agree  **☐** | Agree  **☐** | Neutral  **☐** | Disagree  **☐** | Strongly Disagree  **☐** |

| PR14. | The project was worth the time I spent on it | | | | |
| --- | --- | --- | --- | --- | --- |
|  | Strongly Agree  **☐** | Agree  **☐** | Neutral  **☐** | Disagree  **☐** | Strongly Disagree  **☐** |

**Convenience**

The following three (3) statements are about how convenient it was for you to contribute throughout the project.

| CN1. | I had the opportunity to provide input into selecting my tasks for the project | | | | |
| --- | --- | --- | --- | --- | --- |
|  | Strongly Agree  **☐** | Agree  **☐** | Neutral  **☐** | Disagree  **☐** | Strongly Disagree  **☐** |

| CN3. | Throughout the project, I had sufficient time to complete my tasks for the project | | | | |
| --- | --- | --- | --- | --- | --- |
|  | Strongly Agree  **☐** | Agree  **☐** | Neutral  **☐** | Disagree  **☐** | Strongly Disagree  **☐** |

| CN4. | I had opportunities to express my views | | | | |
| --- | --- | --- | --- | --- | --- |
|  | Strongly Agree  **☐** | Agree  **☐** | Neutral  **☐** | Disagree  **☐** | Strongly Disagree  **☐** |

**Contributions**

The following three (3) statements are about your contributions throughout the project.

| CT1. | I contributed by providing my perspective | | | | |
| --- | --- | --- | --- | --- | --- |
|  | Strongly Agree  **☐** | Agree  **☐** | Neutral  **☐** | Disagree  **☐** | Strongly Disagree  **☐** |

| CT2. | My contributions were a good use of my time | | | | |
| --- | --- | --- | --- | --- | --- |
|  | Strongly Agree  **☐** | Agree  **☐** | Neutral  **☐** | Disagree  **☐** | Strongly Disagree  **☐** |

| CT4. | My workload in the project was manageable | | | | |
| --- | --- | --- | --- | --- | --- |
|  | Strongly Agree  **☐** | Agree  **☐** | Neutral  **☐** | Disagree  **☐** | Strongly Disagree  **☐** |

**Team Environment and Interaction**

The following two (2) statements are about the research environment and interaction throughout the project.

| T2. | I was an equal partner in the research project team | | | | |
| --- | --- | --- | --- | --- | --- |
|  | Strongly Agree  **☐** | Agree  **☐** | Neutral  **☐** | Disagree  **☐** | Strongly Disagree  **☐** |

| T5. | There was trust among the research project team members | | | | |
| --- | --- | --- | --- | --- | --- |
|  | Strongly Agree  **☐** | Agree  **☐** | Neutral  **☐** | Disagree  **☐** | Strongly Disagree  **☐** |

**Support**

The following two (2) statements are about the support provided throughout the project.

| SU1. | I received sufficient support to contribute to the project (for example, orientation, readings, training workshops, webinars) | | | | |
| --- | --- | --- | --- | --- | --- |
|  | Strongly Agree  **☐** | Agree  **☐** | Neutral  **☐** | Disagree  **☐** | Strongly Disagree  **☐** |

| SU2. | Any concerns I had were addressed | | | | |
| --- | --- | --- | --- | --- | --- |
|  | Strongly Agree  **☐** | Agree  **☐** | Neutral  **☐** | Disagree  **☐** | Strongly Disagree  **☐** |

**Feel Valued**

The following two (2) statements are about your feeling of being a valued member of the research team.

| FV1. | The research project team appreciated my contributions | | | | |
| --- | --- | --- | --- | --- | --- |
|  | Strongly Agree  **☐** | Agree  **☐** | Neutral  **☐** | Disagree  **☐** | Strongly Disagree  **☐** |

| FV3. | I was offered sufficient recognition for my contributions (for example, payment, authorship, or gifts) | | | | |
| --- | --- | --- | --- | --- | --- |
|  | Strongly Agree  **☐** | Agree  **☐** | Neutral  **☐** | Disagree  **☐** | Strongly Disagree  **☐** |

**Benefits**

The following three (3) statements are about the benefits of your involvement in the project.

| BE1. | I enjoyed being a part of the project | | | | |
| --- | --- | --- | --- | --- | --- |
|  | Strongly Agree  **☐** | Agree  **☐** | Neutral  **☐** | Disagree  **☐** | Strongly Disagree  **☐** |

| BE2. | I made an impact on the decisions in the project | | | | |
| --- | --- | --- | --- | --- | --- |
|  | Strongly Agree  **☐** | Agree  **☐** | Neutral  **☐** | Disagree  **☐** | Strongly Disagree  **☐** |

| BE4. | My involvement had positive impacts on my life | | | | |
| --- | --- | --- | --- | --- | --- |
|  | Strongly Agree  **☐** | Agree  **☐** | Neutral  **☐** | Disagree  **☐** | Strongly Disagree  **☐** |

Next, there is an optional free text to share your personal story. This will not be linked to your survey responses. You can choose to share your name with your story if you would like. This is your personal decision. Your input will be valued either way.
*[free text box with no word limit]*

# GRIPP2 (guidance for reporting patient and public involvement) long-form checklist

| Section and topic | | Item | Reported on page No |
| --- | --- | --- | --- |
| Section 1: Abstract of paper | | |  |
| 1a: Aim | | Report the aim of the study | 1 |
| 1b: Methods | | Describe the methods used by which patients and the public were involved | 1 |
| 1c: Results | | Report the impacts and outcomes of PPI in the study | 1 |
| 1d:Conclusions | | Summarise the main conclusions of the study | 1 |
| 1e: Keywords | | Include PPI, “patient and public involvement,” or alternative terms as keywords | 1 |
| Section 2: Background to paper | | |  |
| 2a: Definition | | Report the definition of PPI used in the study and how it links to comparable studies | 6 |
| 2b: Theoretical underpinnings | | Report the theoretical rationale and any theoretical influences relating to PPI in the study | 6 |
| 2c: Concepts and theory development | | Report any conceptual models or influences used in the study | 6 |
| Section 3: Aims of paper | | |  |
| 3: Aim | | Report the aim of the study | 8 |
| Section 4: Methods of paper | | |  |
| 4a: Design | | Provide a clear description of methods by which patients and the public were involved | 9 |
| 4b: People involved | | Provide a description of patients, carers, and the public involved with the PPI activity in the study | 9 |
| 4c: Stages of involvement | | Report on how PPI is used at different stages of the study | 9 |
| 4d: Level or nature of involvement | | Report the level or nature of PPI used at various stages of the study | 10 |
| Section 5: Capture or measurement of PPI impact | | |  |
| 5a: Qualitative evidence of impact | If applicable, report the methods used to qualitatively explore the impact of PPI in the study | | 11 |
| 5b: Quantitative evidence of impact | If applicable, report the methods used to quantitatively measure or assess the impact of PPI | | 11 |
| 5c: Robustness of measure | If applicable, report the rigour of the method used to capture or measure the impact of PPI | | 11 |
| Section 6: Economic assessment | | |  |
| 6: Economic assessment | If applicable, report the method used for an economic assessment of PPI | | N/A |
| Section 7: Study results | | |  |
| 7a: Outcomes of PPI | Report the results of PPI in the study, including both positive and negative outcomes | | 14 |
| 7b: Impacts of PPI | Report the positive and negative impacts that PPI has had on the research, the individuals involved (including patients and researchers), and wider impacts | | 13 |
| 7c: Context of PPI | Report the influence of any contextual factors that enabled or hindered the process or impact of PPI | | 14 |
| 7d: Process of PPI | Report the influence of any process factors, that enabled or hindered the impact of PPI | | 14 |
| 7ei: Theory development | Report any conceptual or theoretical development in PPI that have emerged | | 16 |
| 7eii: Theory development | Report evaluation of theoretical models, if any | | N/A |
| 7f: Measurement | If applicable, report all aspects of instrument development and testing (eg, validity, reliability, feasibility, acceptability, responsiveness, interpretability, appropriateness, precision) | | N/A |
| 7g: Economic assessment | Report any information on the costs or benefit of PPI | | N/A |
| Section 8: Discussion and conclusions | | |  |
| 8a: Outcomes | Comment on how PPI influenced the study overall. Describe positive and negative effects | | 16 |
| 8b: Impacts | Comment on the different impacts of PPI identified in this study and how they contribute to new knowledge | | 17 |
| 8c: Definition | Comment on the definition of PPI used (reported in the Background section) and whether or not you would suggest any changes | | Explained that PPIE goes beyond involvement to engagement, which applied to this group but other researchers may refer to PPI |
| 8d: Theoretical underpinnings | Comment on any way your study adds to the theoretical development of PPI | | Success of codesign using person-based approach |
| 8e: Context | Comment on how context factors influenced PPI in the study | | Time and funding are rate-limiting |
| 8f: Process | Comment on how process factors influenced PPI in the study | | As above |
| 8g: Measurement and capture of PPI impact | If applicable, comment on how well PPI impact was evaluated or measured in the study | | Use of validated survey + qualitative analysis of personal narratives, PPI as co-authors |
| 8h: Economic assessment | If applicable, discuss any aspects of the economic cost or benefit of PPI, particularly any suggestions for future economic modelling. | | Recommended robust costings for early and frequent PPI throughout a research project |
| 8i: Reflections/critical perspective | Comment critically on the study, reflecting on the things that went well and those that did not, so that others can learn from this study | | Researcher wrote the first draft of the manuscript. Would be preferable to have PPI to write in own words, may be easier with a in-person workshop or with more funding for a series of online group meetings and time for researcher to have individual communication then collate responses for the group |

# Personal narratives

| **ID** | **Optional personal narrative (free text, in own words)** |
| --- | --- |
| 1 | I felt honoured to be part of the project and was encouraged by the amount of work and enthusiasm shown by those involved in the research and the impact that this will make on the treatment and prevention of cancer. Although some of the medical terminology and use of acronyms could be confusing in some of the presentations, I have learnt a great deal from participating in the project and felt that I was listened to and valued during the discussions. |
| 2 | I got involved with the Patient Reference Panel after being diagnosed with cancer due to Lynch syndrome. My own experience with cancer, along with the support I received through and beyond genetic testing, motivated me to join the CanGene-CanVar programme, hoping to make a difference for others in similar situations. Over the past five years, working with the research team has been incredibly rewarding. I’ve had the opportunity to contribute in various ways—attending in-person meetings, presenting, and collaborating on the design and facilitation of focus groups. I’ve also really enjoyed networking with others and helping to shape Lynch Choices, a highly valuable resource for people diagnosed with Lynch syndrome. |
| 3 | Being part of the CanGene-CanVar PPI team has been one of the best projects that I have taken part in. I joined the team after my own cancer journey and subsequent genetic diagnosis of Lynch Syndrome. Everybody has their own story and each is as important as the next and this is exactly the way I was made to feel as part of the PPI panel. The whole panel were so nice, they all listened to each person’s story, each person’s opinions and ideas and I think I speak for everyone in that we were all made to feel like we mattered and that there was a way that we could help other people that may find themselves in the same situation and I feel this is something that is such a healing process. It made me feel like I wasn’t alone, I didn’t have much confidence at first but I was always encouraged to speak and to engage in discussions. I still have a way to go in that regard but I do feel much more confident than I was. I have been given opportunities to publicly speak, to write blog posts and to also record a podcast. I even spent a week with the ethics group in Oxford and travelled to Vilnius in Lithuania. I will definitely be sad to see the end of this project but I have come away with so much knowledge and made some epic friends. Most of all, for me, I feel like in some small way if I can help one person on their journey, every minute is worthwhile. |
| 4 | I have been involved with patient advocacy for over 15 years, and I can honestly say that working with the CanGene CanVar research team was a privilege and an absolute pleasure. As a patient group we were kept informed and listened to throughout. Our opinions were valued and acted upon which is not always the case. The work carried out is a rare example of true co-design. As patients we felt useful and an integral part of the team. |
| 5 | It is so long since I made a contribution to the project that I have answered 'Neutral' to many questions. However, it has been such a well-run project from the PPI perspective that I didn't feel I was needed! |
| 6 | I am incredibly proud of being a patient rep for CanGene-CanVar, everything it has achieved and how it has involved patients in the achievement of it. It has been a fantastic opportunity to get patient voice into decision making and ensuring that decisions included consideration of the impact on patients and their needs. Not only was my lived experience considered, it was respected. I also had the opportunity to learn from the academics we worked with and their research findings as well as meet some brilliant people, including my co patient reps who I hope to continue to work with in the future. |
| 7 | Limited support and information for my family (including young son) when I was subject to bowel cancer, surgery and chemo (over 10 years ago), my goal was to help change this for others experiencing the same life journey. Happy to help. |
| 8 | The goal of the project was clear at the beginning. This gave a clear focus to what needed to be achieved. The researchers were very open to input from the Patient Panel. With a range of different patient representatives, it wasn’t possible to incorporate all views as sometimes they were not aligned. The researchers spent time explaining what changes they had implemented and why in various drafts as we went through the iterative process of design. I was made to feel valued, not just by being offered compensation but by being offered the opportunity to be a co-author on papers. For me the latter is more important. |
| 9 | In September 2019, during my undergraduate degree when I was living in Spain for my ERASMUS year, I got a call from my Dad to tell me he had been diagnosed with Pancreatic Cancer. My world was turned upside down in that moment, but I had no idea quite how much it would change my life. In July 2020, my Dad told myself and my two sisters that he had tested positive for the BRCA2 gene mutation, meaning that we would need to get tested also. In October 2020, I found out that I am a carrier of the mutation.  I did not have good experiences with genetic counselling. I was only 21, and they kept reminding me of that. I knew I wanted to have surgery, as I had watched my Dad go through cancer treatment and I wanted to reduce my own risk as much as possible. When I said I wanted to have a mastectomy, and that I had sought the opinions of a breast surgeon, I was borderline reprimanded and told I was “too young to be making those kinds of decisions”. What they didn’t account for was that dealing with a close family member with cancer and a BRCA diagnosis forces you to grow up. It was clear that they saw my age on a form or piece of paper, rather than viewing me as a whole person. I had a bilateral risk-reducing mastectomy with immediate reconstruction in May 2023, when I was 23 years old, so far in my life, it is the best decision I have ever made. After my experiences with genetic counselling, I decided I wanted to help in some way. I joined the Patient Reference Panel as part of this. I was keen to, as far as I could, try to ensure that someone else in my position wouldn’t be told the same, or something similar. I feel that the way in which we present choices to patients, and give them the information to make informed decisions, is so important. I have really valued the sense of voice and agency that the PRP has given me. |
| 10 | Although I don’t have Lynch Syndrome or a known BRCA gene, I have lived through breast cancer, experiencing both DCIS and invasive cancer. I underwent aggressive treatment including mastectomy, reconstruction, chemotherapy, radiotherapy, Herceptin, and long-term hormone therapy with Tamoxifen. This treatment pathway later led to endometrial hyperplasia, which meant I had to have a full hysterectomy and bilateral oophorectomy – all before the age of 50, while raising a young family and caring for elderly parents. This personal journey made me deeply aware of the emotional, physical, and practical complexities of making cancer-related decisions. It’s why I was so committed to the CanGene-CanVar decision aid project. Even though my specific diagnosis wasn’t linked to inherited cancer risk, I understood the burden and uncertainty patients carry — and the need for clear, compassionate, co-designed tools to support their choices. Being involved as a public contributor felt meaningful and purposeful, and I was proud to be treated as an equal partner throughout. |
| 11 | Being a partner in the co-design of Lynch Choices has been a truly rewarding experience. It gave me the chance to contribute lived insights and perspectives that shaped something meaningful and tailored to the needs of real people and families. I felt respected and valued throughout the process—our ideas weren’t just heard; they genuinely informed decisions. It’s been powerful to see how collaboration between professionals and those with lived experience can create something so much more impactful and human. |
| 12 | As a patient advocate for people who are affected by Lynch Syndrome in Ireland, being on the ground with families as they navigate their diagnosis and seeing the repeated difficulties faced in obtaining timely information and supports needed to aid complex decision making when contending with fragmented pathways & services, and conflicting information, having the opportunity to contribute to this body of work - CanGene-CanVar decision aid codesign project, the creation of an easily accessible, trustworthy and practical aid that meets the information and support requirements of all who use it, both patients and clinicians alike, was like winning the lotto to be honest. From first introductions to me writing this, and everything in-between, it's been the most rewarding and I daresay fun experience. The genuine dedication of the research team to truly listen to and understand the experiences and needs of people living with Lynch and to work together to co- design and develop a resource that would help lessen their burdens has been truly heartwarming. It's great to have such comprehensive information across the many relevant LS topics available on a single platform that's intuitive to individual needs. Kelly and the research team have been incredibly supportive, empowering and professional throughout, treating me as a valued team member whose contributions are important. It was wonderful to see my thoughts and suggestions shape the project from beginning to end- this wasn't a box ticking exercise; this was meaningful partnership. The level of involvement and communication throughout, so brilliantly guided by Kelly, created a culture of trust, respect and comradery. We made a great team and patients will benefit from what we've all achieved by working together. I'm extremely grateful to Kelly and every person involved in this project for everything they do to improve the lives of people living with Lynch syndrome. The whole affair was a masterclass in PPI - empathy, inclusion and science communication at its finest, and a pleasure to be part of. |
| Researcher | The Patient Reference Panel and public partners for the CanGene-CanVar project to co-design a decision aid were incredibly engaged, dedicated and supportive of each other and of me. As a (mature) PhD student, I valued the opportunity to take a step back from clinical practice where for many years I had been responsible for the care of individuals. Consultations were often time pressured and focussed on a particular decision. Adopting the positionality of a researcher allowed me to seek a more holistic view of the views and priorities of the patient population by listening, remaining curious and open to learning new things. From the start it was clear that I shared aims and enthusiasm with the patient and public partners to improve care and experiences. Enthusiasm from the partners gave me great motivation and satisfaction. We formed a trusting relationship that brought many benefits, informing the direction and outcomes of my research as well as my clinical practice. Having a small core group that met many times, including in person, helped to develop trust, mutual respect and foster productive partnerships. Partners often disagreed, but this was handled respectfully, with an openness to consider other views. It felt like there was never enough time or resource to learn as much as I would have liked from them. At the end of the project, I was filled with many ideas and inspirations about future research questions. It was clear to me throughout that the patient and public partners cared about the research aims and were incredibly generous with their time and energy to help others, despite juggling many demands in their social worlds and sometimes dealing with health issues as well. I recommend any future researchers carefully reflect on the value of PPIE starting from the conception of their research idea. Robust funding and time allocation should be requested from funders to fully realise the potential benefits of patient and public partnerships in research. |
